# How to Interpret Long‐Term Prognostic Impact of Catheter Ablation for Electrical Storm

**DOI:** 10.1002/clc.70301

**Published:** 2026-04-11

**Authors:** Naoya Kataoka, Teruhiko Imamura

**Affiliations:** ^1^ Second Department of Internal Medicine University of Toyama Toyama Japan

**Keywords:** cardiogenic shock, heart failure, hemodynamics

To the Editor,

While the number of catheter ablation procedures performed for refractory electrical storm has been increasing, their long‐term clinical impact remains uncertain. In the present study, the authors demonstrated that ventricular tachycardia (VT) ablation was safe and effective for managing electrical storm, providing high acute procedural success and allowing most patients to be discharged [1]. Conversely, these patients continued to face high risks of long‐term morbidity and mortality. Several concerns have been raised.

The retrospective single‐arm design without a control group introduces major selection bias [1]. Patients who experienced an electrical storm but did not undergo VT ablation were excluded from analysis, and their clinical outcomes remain unknown. The inclusion of a comparator cohort treated with medical therapy alone—regarding survival, end‐organ function, requirement for mechanical circulatory support or ventilation support, re‐hospitalization, and VT recurrence—would further clarify the true clinical implication of VT ablation in this population [2].

Most participants had heart failure with reduced ejection fraction, and lower left ventricular ejection fraction was associated with worse long‐term outcomes following VT ablation [1]. Given that guideline‐directed medical therapy (GDMT) for heart failure has a significant impact on improving cardiac function and prognosis [3], the prescription rate and optimization of GDMT should be considered when interpreting the effect of VT ablation. Whether post‐ablation medical therapy was optimized and whether GDMT intensification influenced clinical outcomes remains unclear.

VT recurrence continued to occur soon after the ablation procedure in the present cohort [1], despite the general notion that the cumulative incidence of VT recurrence tends to plateau during the first year after ablation [4]. This early recurrence pattern may suggest that the enrolled patients were more critically ill than conventional ablation cohorts, or that urgent ablation during electrical storm might not always achieve sufficient substrate modification. More detailed baseline information—such as hemodynamic severity, extent of myocardial scar, refractory arrhythmia burden before the procedure, and requirement for vasopressors or temporary mechanical support—would help clarify the clinical severity of this population.

The study highlights that successful VT ablation does not fully mitigate long‐term risks in patients with electrical storm [1]. This condition often reflects advanced structural heart disease, and aggressive post‐ablation management—including heart failure optimization, candidacy assessment for ventricular assist devices or transplantation, and structured follow‐up—may be essential to improve outcomes. Future studies should explore whether integrating substrate‐based ablation strategies with comprehensive multidisciplinary heart failure care can further improve survival.

## Funding

The authors have nothing to report.

## Ethics Statement

The authors have nothing to report.

## Consent

The authors have nothing to report.

## Conflicts of Interest

The authors declare no conflicts of interest.

## Data Availability

The authors have nothing to report.
